# Supplementary material for: Cell-free prediction of protein expression costs for growing cells
Source: Nat Commun. 2018 Apr 13;9:1457. doi: 10.1038/s41467-018-03970-x (PMC5899134; doi:10.1038/s41467-018-03970-x)
Supplement: Supplementary file 1 — Supplementary Information [file 41467_2018_3970_MOESM1_ESM.pdf]

Supplementary Information

Cell-free prediction of protein expression costs for growing cells

Borkowski *et al.*

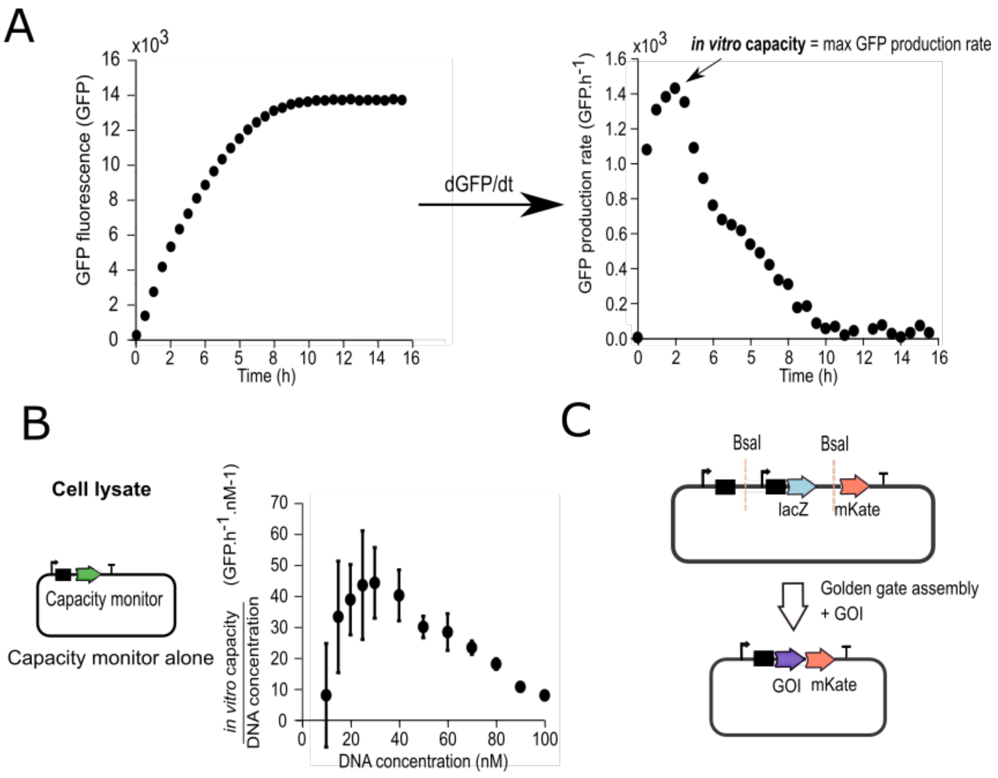

Supplementary Figure 1: Cell lysate measurements and backbone design (**A**) The graph on the left is an example of the GFP fluorescence measurements in cell lysate as a function of time using 30 nM of the capacity monitor plasmid cell lysate mix. The graph on the right is the derivate of the GFP fluorescence, *i.e.* the GFP production rate. GFP production rates were calculated with: GFP production rate at t2 = [total GFP(t3) – total GFP(t1)]/(t3 – t1) with t2 = time of the measurement, t3 = t2 + 0.25 hr and t1= t2 – 0.25 hr. The *in vitro* capacity is the maximum value of GFP production rate. (**B**) Measured *in vitro* capacity divided by DNA concentration with the capacity monitor plasmid added at different concentrations in a 10.5 µl cell lysate mix. Error bars show standard error of three independent repeats. (**C**) The entry vector design for this work consists of a promoter BBa_J23106, the BCD 2 or BCD 21 or RBS B0034, a *bsa*I restriction site (`ggtctcccatt`), promoter pLac, lacZ, a *bsa*I restriction site, linker sequence (`aggtctcagctt`), codon optimised mkate and terminator. Golden Gate assembly was used to integrate different GoIs leading to GoI-mKate fusion proteins.

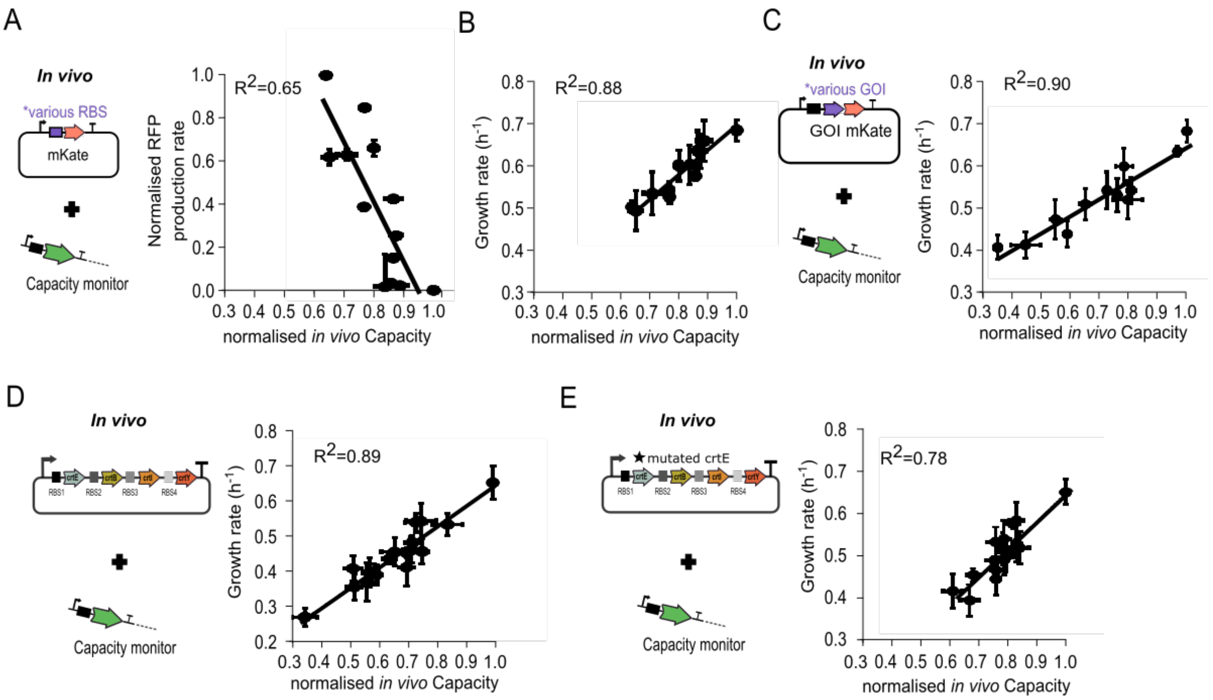

Supplementary Figure 2: *In vivo* correlation between Normalized Capacity, Normalised RFP production rate and Growth rate. (**A**) Correlation between normalized RFP production rates and normalized *in vivo* capacity with DH10B cells. Constructs expressing mkate with different strength RBS sequences (same strains as Figure 1E). (**B, C, D & E**) Correlation between growth rates and normalized *in vivo* capacity with DH10B cells (**B**) Constructs expressing mKate with different strength RBS sequences (same strains as Figure 1E). (**C**) Constructs with various genes of different sizes paired with RBS BCD2 and fused to mKate (same strains as Figure 1F). (**D**) Beta-carotene biosynthesis operons without an inactivating mutation in the crtE gene (same strains as Figure 4B left). (**E**) Beta-carotene biosynthesis operons with an inactivating mutation in the crtE gene (same strains as Figure 4B right). Error bars show standard error of three independent repeats. Values are normalised to the capacity obtained with capacity monitor plasmid alone.

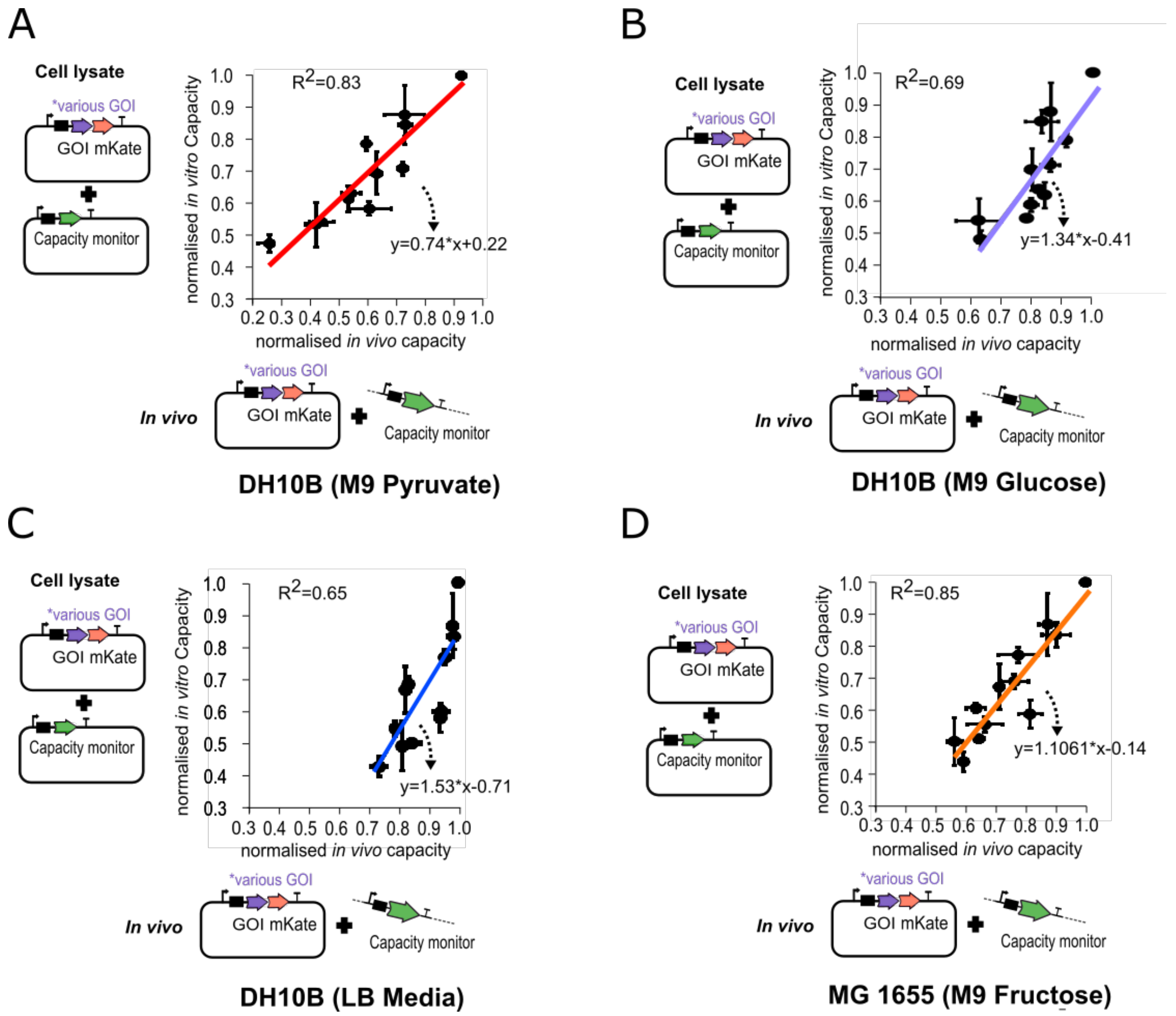

Supplementary Figure 3: Medium-dependent burden. (**A,B,C,D**) Correlation between normalized *in vitro* capacity measured in cell lysate (same value as Figure 1E) and normalized *in vivo* capacity measured in DH10B (for A,B and C) or MG1655 (for D) using constructs with various genes of different sizes fused to mKate and paired with RBS BCD2. All *in vivo* measurements have been done in the conditions described in Figure 1G and H: (A) DH10B in minimal media M9 + 0.5% pyruvate, (B) DH10B in minimal media M9 + 0.5% Glucose, (C) DH10B in LB, (D) MG1655 in minimal media M9 + 0.5% fructose. Error bars show standard error of three independent repeats. Values are normalised to the capacity obtained with capacity monitor plasmid alone.

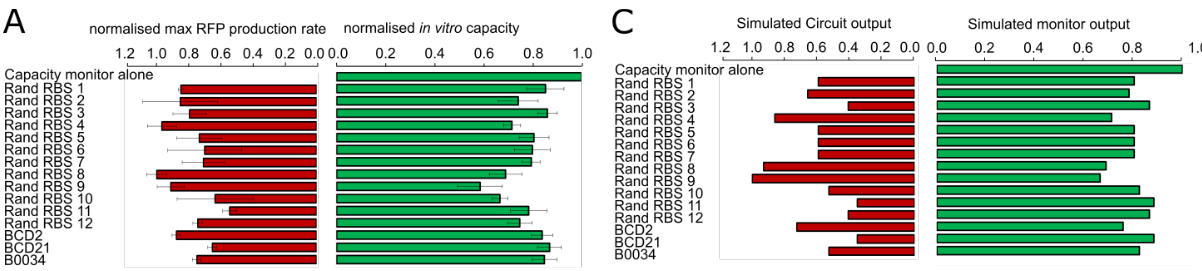

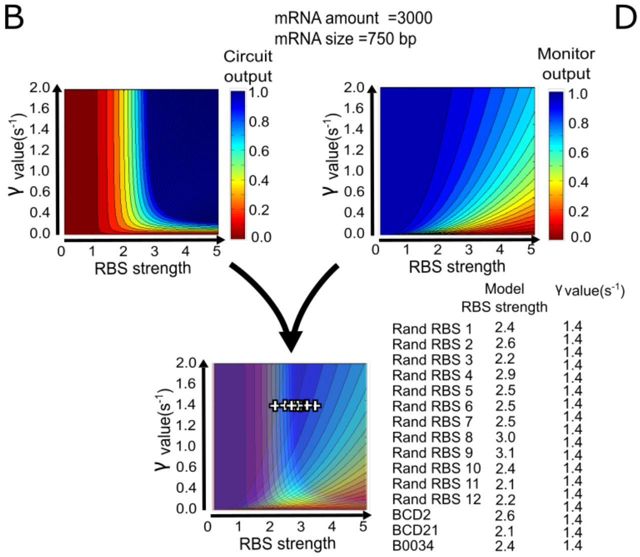

D

RBS name	Prediction RBS calculator	**Model RBS Strength**
**Rand RBS1**	1660.1	**2.4**
**Rand RBS2**	769.0	**2.6**
**Rand RBS3**	420.3	**2.2**
**Rand RBS4**	8823.9	**2.9**
**Rand RBS5**	2090.3	**2.5**
**Rand RBS6**	167.2	**2.5**
**Rand RBS7**	39.3	**2.5**
**Rand RBS8**	9252.2	**3.0**
**Rand RBS9**	4610.0	**3.1**
**Rand RBS10**	219.1	**2.4**
**Rand RBS11**	219.1	**2.1**
**Rand RBS12**	56.8	**2.2**
**BCD2**	19700.6	**2.6**
**BCD21**	1051.2	**2.1**
**B0034**	3002.0	**2.4**

Supplementary Figure 4: Cell lysate measurements and simulated output and capacity at steady-state determined by a translational resource model. (**A**) Normalised max RFP production rate and normalised *in vitro* capacity measurements of constructs with mKate alone under control of different RBS/BCDs (library from Figure 1E, supplementary table 1). Normalised max RFP production rates are the max RFP production divided by the mean max RFP production of the Rand RBS 8 construct (strongest RBS from the collection). Error bars show standard error of three independent repeats. (**B**) Simulation of the construct output (corresponding to the normalised max RFP production rate measurement) and monitor output (corresponding to the normalised *in vitro* capacity measurement). The construct and monitor outputs graphs have been merged to deduce the RBS strengths and γ value of the mKate construct library. All the constructs produce the same mKate protein and thus have the same γ value. (**C**) Results of the simulation of panel B. (**D**) Comparison between the RBS calculator predictions and our model simulations. The order of RBS strength is respected when compared between RBS Calculator predictions and our model simulations. BCD2 strength seems to be underestimated in our model compare to the RBS Calculator. Except for BCD2, our simulations reveal that the strongest RBS of our collection has a RBS strength=3.0, while medium strength RBSs have an RBS strength around 2.5, and the weakest RBS has an RBS strength=2.0.

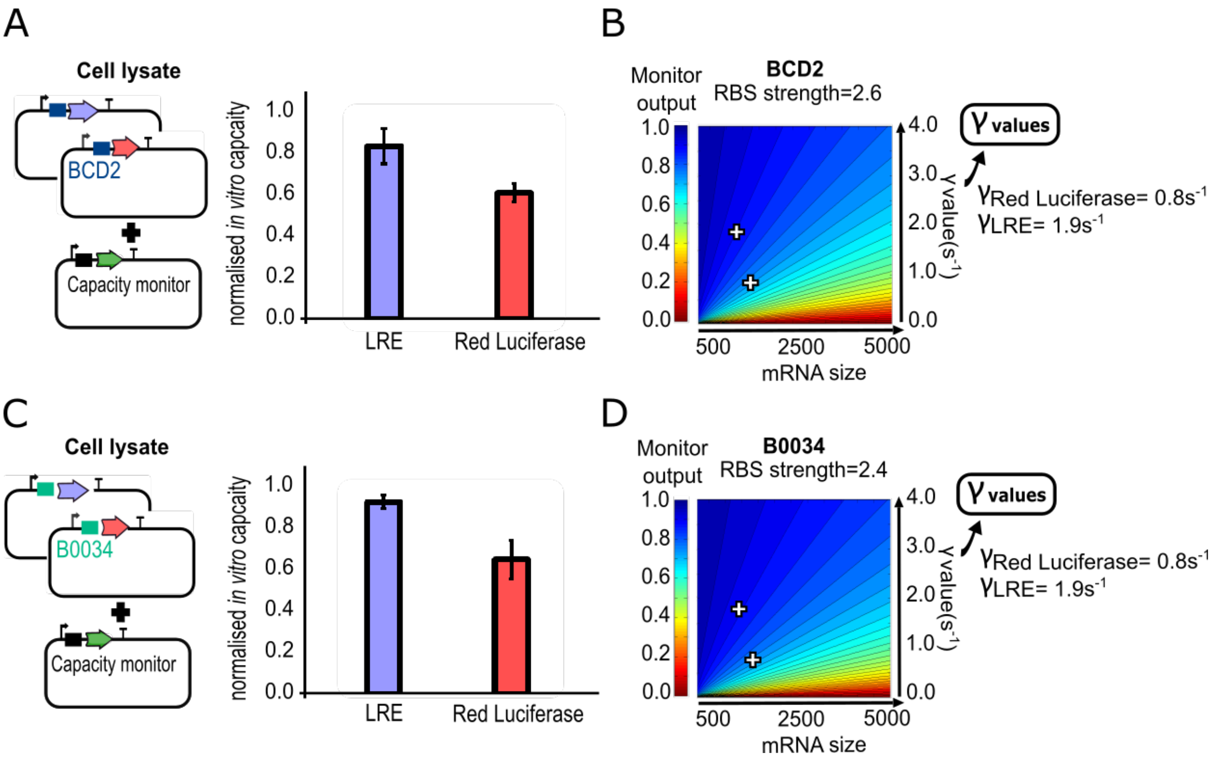

Supplementary Figure 5: Competition for resources in cell lysate between the enzymes of the luciferase pathway and the capacity monitor. (**A**) Normalised *in vitro* capacity measured with capacity monitor and the LRE or Red luciferase (under control of BCD2) plasmids added in the cell lysate mix. (**B**) Heat maps of simulated capacity (monitor output) when mRNA size and the γ value of a synthetic construct are varied as RBS strength is fixed. (**C,D**) Similar experiments as panels A, B but with LRE or Red luciferase under control of the RBS B0034. Values are normalised to the capacity obtained with capacity monitor plasmid alone.

A

Construct name	Promoter Name	Model mRNAs Strength
Luci_J23	P5-J23100	600
Luci_1	P5-J23100	600
Luci_2	P5-J23100	600
Luci_3	P5-J23100	600
Luci_4	P5-J23100	600
Luci_5	P5-J23100	600
Luci_6	P5-J23100	600
Luci_7	P5-J23100	600
Luci_8	P5-J23100	600
Luci_9	P5-J23100	600
Luci_10	P5-J23100	600
Luci_11	P5-J23100	600
Luci_12	P5-J23100	600
Luci_13	P5-J23100	600

Prediction RBS calculator	Model RBS1 Strength
12130.0	3.0
308.6	2.5
159.6	2.5
26.3	2.0
101.5	2.0
12130.0	3.0
769.4	2.5
12130.0	3.0
118.1	2.0
95.2	2.0
6.7	2.0
7.79	2.0
91.26	2.0
536.74	2.5

Prediction RBS calculator	Model RBS2 Strength
606357.6	3.0
10586.9	2.0
606357.6	3.0
606357.6	3.0
43557.3	2.0
254806.2	2.5
767273.6	3.0
78884.9	2.5
362.0	2.0
16928.2	2.0
333796.0	2.5
3503.72	2.0
691.52	2.0
90287.85	2.5

B

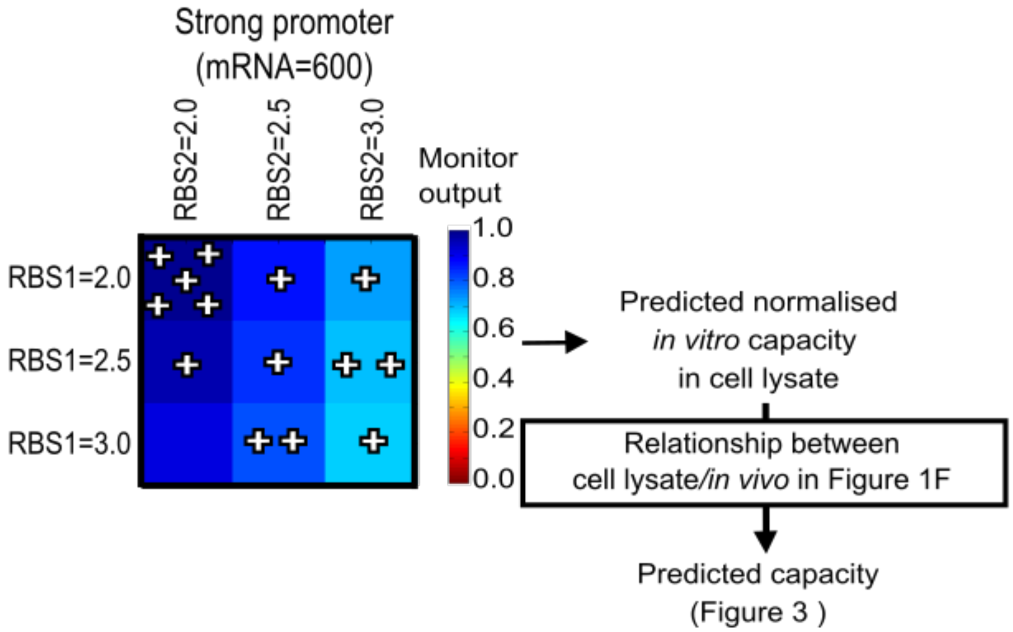

Supplementary Figure 6: Burden prediction for luciferase operons. (**A**) RBS Calculator prediction using the operon calculator. We ranked the model RBS values as described in Supplementary Figure 4D. (**B**) Predictions for the competition between the capacity monitor and the 2 luciferase enzymes were performed using the γ values obtain in Supplementary Figure 5 and the RBS values obtain in panel A.

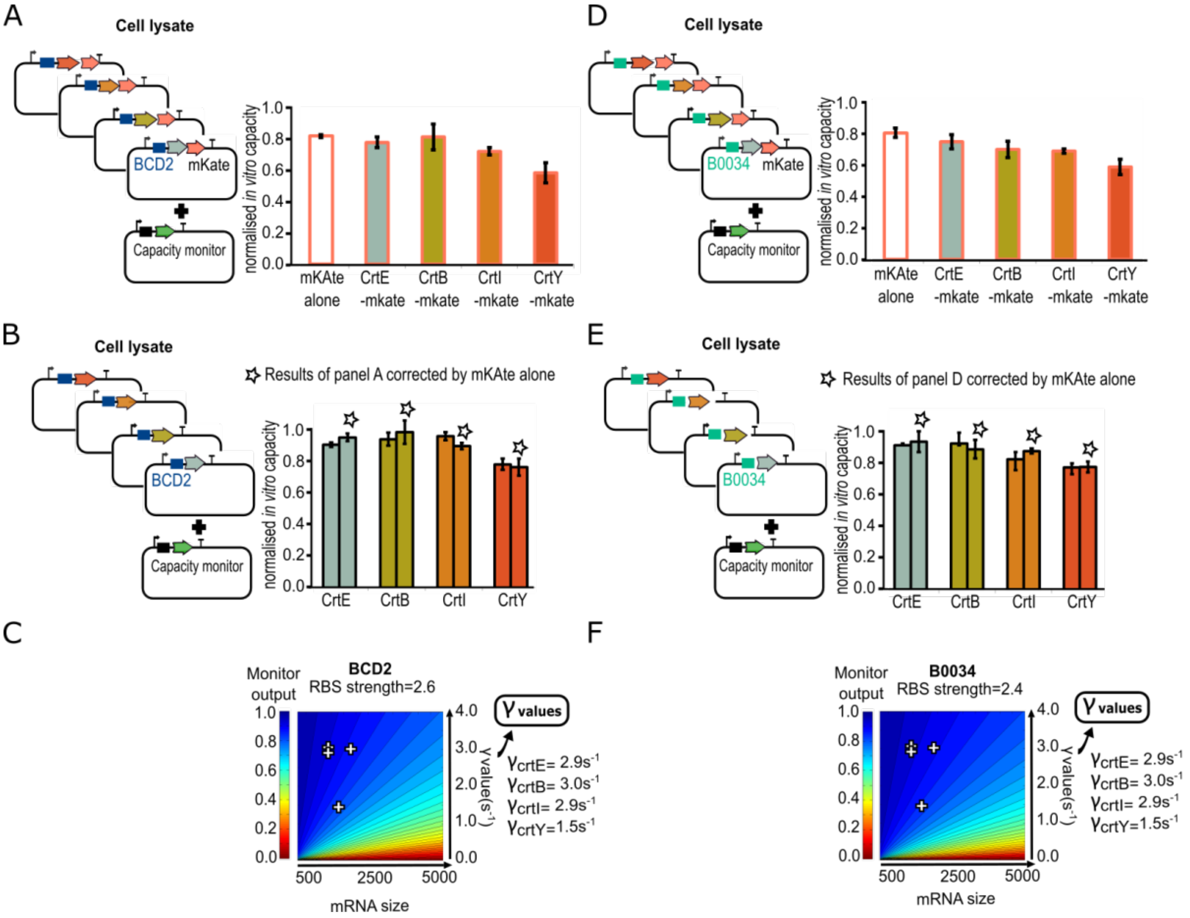

Supplementary Figure 7: Competition for the resources in cell lysate between the enzymes of the beta-carotene pathway and the capacity monitor. (**A**) Normalised *in vitro* capacity measured with capacity monitor and the crt-mKate fusion constructs added in the cell lysate mix. Crt are the enzymes of the beta-carotene pathway. The production of mKate (mKate alone) under control of BCD2 leads to a competition for the resources as the normalised *in vitro* capacity is lower than 1. (**B**) Normalised *in vitro* capacity measured with capacity monitor and the crt enzymes in the cell lysate mix. The mKate CDS has been removed to determine the competition for resource due to the crt enzymes alone. The normalised *in vitro* capacity of the crt-mKate fusions were corrected by removing the cost of expressing mKate alone taken from the data from panel A. The normalised *in vitro* capacity of the crt enzymes and of the corrected crt-mKate exhibit similar values showing that both construct backbone with mKate fusion and without mKate are safe to use for γ value calculation. (**C**) Heat maps of simulated capacity (monitor output) when mRNA size and the γ value of a synthetic construct are varied as RBS strength is fixed (the BCD2 RBS strength has been obtained as explained in Supplementary Figure 4). (**D, E, F**) Similar experiment as panels A, B, C but with RBS B0034. Values are normalised to the capacity obtained with capacity monitor plasmid alone.

A

Construct name	Promoter name	Model mRNA Strength	Prediction Operon calculator	Model RBS Strength	Prediction Operon calculator	Model RBS Strength	Prediction Operon calculator	Model RBS Strength	Prediction Operon calculator	Model RBS Strength
A4-2	P7-J23113	60	2.6	2.0	1891	2.5	1040	2.0	839	2.5
B3-1	P7-J23113	60	232.3	3.0	2457	3.0	47664	3.0	850	2.5
B4-1	P7-J23113	60	345.2	3.0	3498	3.0	32075	3.0	911460	3.0
B8-1	P7-J23113	60	7.9	2.5	1579	2.5	90441	3.0	44	2.0
B10-2	P7-J23113	60	0.3	2.0	671	2.0	390	2.0	6783	3.0
C1-1	P7-J23113	60	1.8	2.0	2911	3.0	270383	3.0	4027	3.0
C7-1	P7-J23113	60	0.0	2.0	3082	3.0	7264	2.5	154	2.0
D2-1	P7-J23113	60	13.6	2.5	932	2.0	187	2.0	1025	2.5
D6-1	P7-J23113	60	16.3	2.5	771	2.0	63725	3.0	808	2.5
B7-2	P6_J23106	300	0.2	2.0	3096	3.0	3540	2.0	1267	2.5
C6-1	P6_J23106	300	1.4	2.0	2474	3.0	6994	2.5	1117	2.5
C9-1	P6_J23106	300	0.1	2.0	814	2.0	1802	2.0	704	2.5
D9-2	P6_J23106	300	16.3	2.5	1378	2.5	194	2.0	4177	3.0
A8-2	P5-J23100	600	15.8	2.5	4418	3.0	13957	2.5	532	2.5
B2-1	P5-J23100	600	5.1	2.5	1103	2.5	194	2.0	2332	3.0
B3-1-str	P5-J23100	600	232.3	3.0	2457	3.0	47664	3.0	850	2.5
B10-2-str	P5-J23100	600	0.3	2.0	671	2.0	390	2.0	6783	3.0

B

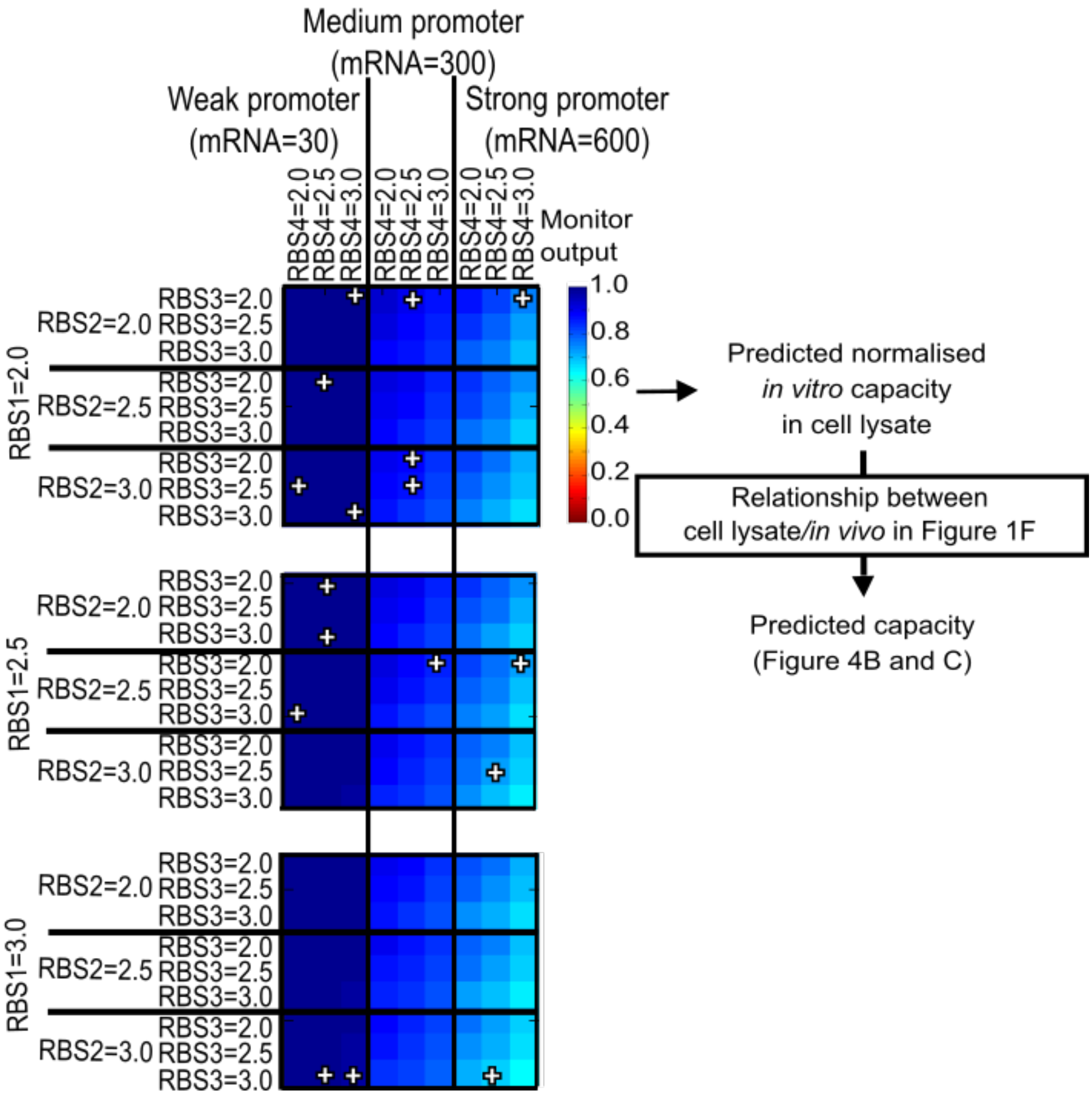

Supplementary Figure 8: Burden prediction for beta-carotene operons. (**A**) RBS Calculator predictions using the operon calculator. We ranked the model RBS value as described in Supplementary Figure 4D. (**B**) Predictions from the competition between the capacity monitor and the 4 *crt* enzymes using the γ values obtained in Supplementary Figure 7 and the RBS values obtain in panel A.

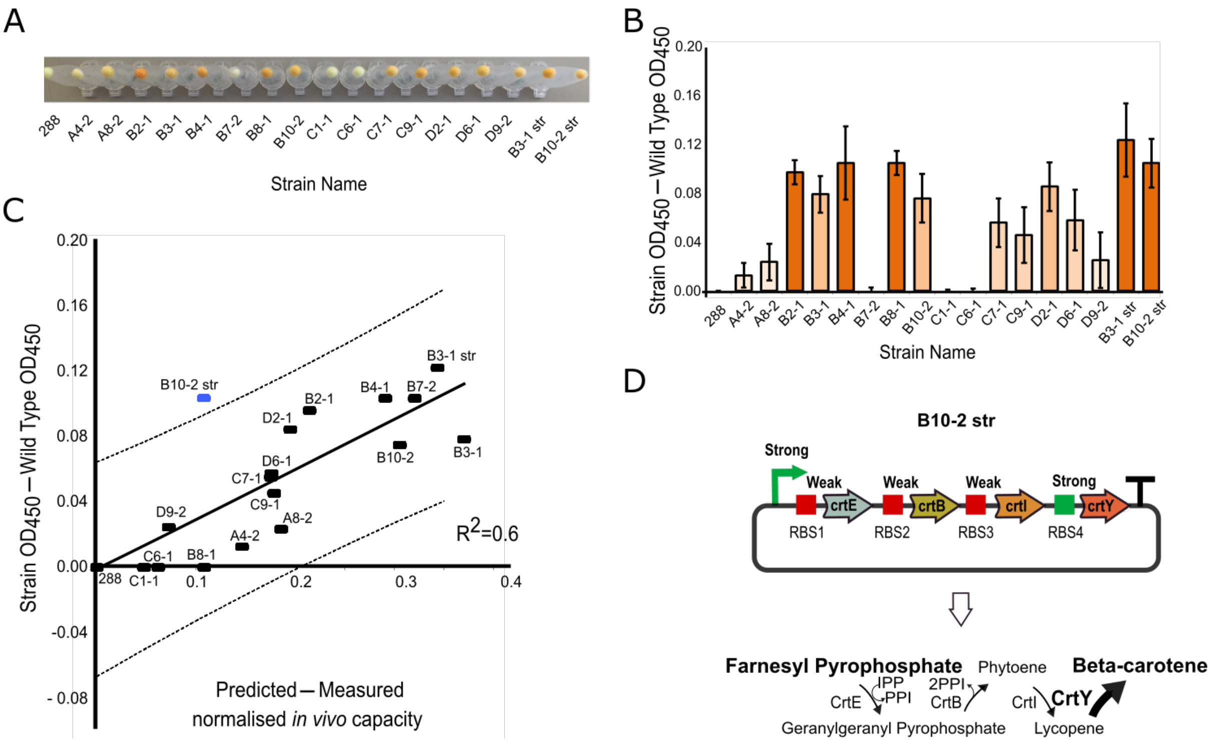

Supplementary Figure 9: Beta-carotene biosynthesis. (**A**) Pellets of the strains producing beta-carotene alongside the names of constructs of the beta-carotene operon library. (**B**) Beta-carotene values were obtained via OD_{450} measurements after acetone treatment. (**C**) Comparison between the beta-carotene production (panel B) and the difference between the predicted and measured *in vivo* capacity (Figure 4C). The dotted lines represent the 95% confident interval. B10-2 str appears to be an outlier (blue dash). (**D**) Diagram of the beta-carotene pathway of the construct B10-2 str. B10-2 str is a construct with a strong promoter, weak RBS sequences for genes 1 to 3 and a strong RBS for gene 4 (Supplementary Figure 8A). This construct should produce a higher amount of CrtY, the last enzyme of the beta-carotene operon, than CrtE, CrtB and CrtI. Values are normalised to the capacity obtained with capacity monitor plasmid alone.

Supplementary Table 1: Library of BCD/RBS sequences

Name	Sequence*
Rand RBS1	GCAAGGGGATAGTG
Rand RBS2	GCAAGGGATTGATA
Rand RBS3	GCAAAGGGAAAGATGG
Rand RBS4	GCAAGGGGGGGGTT
Rand RBS5	GCAAGGGAGGGCGCT
Rand RBS6	GCAAGGGGCGGGGT
Rand RBS7	GCAAGGGCGTATG
Rand RBS8	GCAAAGGGGGTGCGTC
Rand RBS9	GCAAGGGGGCGGGGG
Rand RBS10	GCAAGGGTCGTCT
Rand RBS11	GCAAGGGGGGGTGT
Rand RBS12	GCAAGGGTCGAGT
BCD2	GCAAGGGCCCAAGTTCACTTAAAAAGGAGATCAACAATGAAAGCAATTTT CGTACTGAAACATCTTAATCATGCTAAGGAGGTTTTCT
BCD21	GCAAGGGCCCAAGTTCACTTAAAAAGGAGATCAACAATGAAAGCAATTTT CGTACTGAAACATCTTAATCATGCGAGGGATGGTTTCT
B0034	AAAGAGGAGAAA

* all the RBS/BCD sequences are preceded by the promoter BBa_J23106 (TTTACGGCTAGCTCAGTCCTAGGTATAGTGCTAGCGCAA) and followed by the *mkate* sequence (ATGGAACTGATTAAAGAAAACAT...)

Supplementary Table 2: Library of arbitrarily selected Genes of Interest (GoI)

Name	Function	Size (bp)	Organism
empty	-	0	
Viob_truncated3	(truncated protein)	500	*C. violaceum*
suhB	Inositol-1-monophosphatase (use for the production of glucaric Acid)	804	*E. coli*
URA3	Orotidine 5'-phosphate decarboxylase (ODCase), catalyzes the synthesis of pyrimidine ribonucleotides	820	*S. cerevisiae*
Viob_truncated2	(truncated protein)	1000	*C. violaceum*
Leu2	3-Isopropylmalate dehydrogenase	1111	*S. cerevisiae*
aspA	Aspartate ammonia-lyase	1479	*E. coli*
atf1	Alcohol acetyltransferase	1572	*S. cerevisiae*
Viob_truncated1	(truncated protein)	2000	*C. violaceum*
ste12	Pheromone-responsive transcriptional activator.	2070	*S. cerevisiae*
Viob	Involved in dTDP-N-acetylviosamine synthesis, violacein pathway	2994	*C. violaceum*

Supplementary Note 1: Model overview

This model is based on the Algar *et al.*[1] model of gene expression. The purpose of the model is to capture at first order the competition for resources between a synthetic gene or construct and the capacity monitor. The model focuses on translation, which accounts for the majority of resource usage in fast dividing organisms [2,3,4,5]. The model was originally designed as a ribosome flow model to simulate how ribosomes are removed from the free pool available for translation as they elongate along an mRNA performing translation. Due to features in the coding sequences, this elongation can proceed fast or slow at any step (**Supplementary Figure 10**).

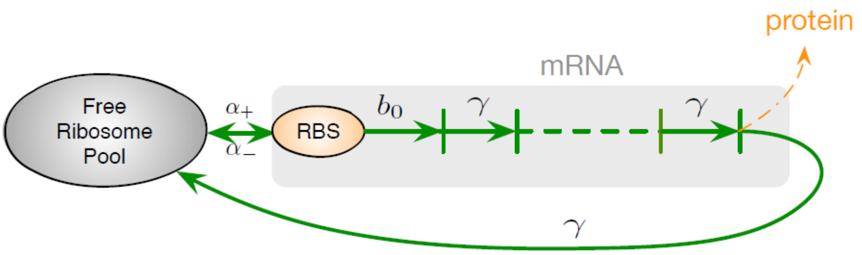

Supplementary Figure 10: Ribosome flow model for competititive translation. Diagram and model are adapted from Algar *et al.*[1]

However, in this work we recognise that measuring or predicting the speed of all elongation steps is not currently feasible, and so the model is modified to collapse all elongation steps into a single lumped parameter, γ, that represents the efficiency of all steps of translation after translation initiation. This lumped parameter takes into account all aspects of translation, not just ribosome availability.

The model acts by simulating a situation where a synthetic construct mRNA competes for a fixed concentration of ribosomes (2500 nM) with the standard capacity monitor mRNA. The capacity monitor is characterised by 4 parameters: mRNA concentration = 900 nM, mRNA

size = 720 bp, RBS strength = 1 and γ = 1 s^{-1} ("translation resources consumed".s^{-1}). The RBS strength impacts the binding rate ($a_+ = a_{+M}$*RBS strength), unbinding rate ($a_- = a_{-M}$/RBS strength) and the first synthesis rate ($b_0 = b_{0M}$*RBS strength), where a_{+M}= 0.0001 rib^{-1} RBS^{-1} s^{-1}, a_{-M}= 200 rib-RBS^{-1} s^{-1} and b_{0M}= 1 s^{-1}.

The model consists of n+3 variables (n = size mRNA / 30) per synthetic gene: G = the average concentration of free ribosomes, Y_0 = the average concentration for ribosomes bound to the RBS across all mRNAs of the synthetic gene, Y_n= the average concentration of ribosomes (across all mRNAs) in position *n*, *mRNA* = the average concentration of mRNA transcripts in the cytoplasm. The size of mRNAs is divided into 30 bases as we assume that the ribosome physical size prevents ribosomes getting closer than 30 bases from each another when on an mRNA.

The kinetics of these variables are described by the following set of ordinary differential equations:

$$\frac{dG}{dt} = -mRNA.a_+.G.\left(1 - \frac{Y_0}{mRNA}\right) + a_-.Y_0 + \gamma.Y_n$$

$$\frac{dY_0}{dt} = mRNA.a_+.G.\left(1 - \frac{Y_0}{mRNA}\right) - a_-.Y_0 - b_0.Y_0.(1 - \frac{Y_1}{mRNA})$$

$$\frac{dY_1}{dt} = b_0.Y_0.(1 - \frac{Y_1}{mRNA}) - \gamma.Y_1.(1 - \frac{Y_2}{mRNA})$$

$$\frac{dY_2}{dt} = \gamma.Y_1.(1 - \frac{Y_2}{mRNA}) - \gamma.Y_2.(1 - \frac{Y_3}{mRNA})$$

$$\cdots$$

$$\frac{dY_n}{dt} = \gamma.Y_{n-1}.(1 - \frac{Y_n}{mRNA}) - \gamma.Y_n$$

$$\frac{dProtein}{dt} = \gamma.Y_n$$

Based on the model, the translation initiation rate is the mean rate of arrival of ribosomes in position 1. It is equal to $b_0.Y_0.(1-\frac{Y_1}{mRNA})$ a part of the equation $\frac{dY_1}{dt} = b_0.Y_0.(1-\frac{Y_1}{mRNA}) - \gamma.Y_1.(1-\frac{Y_2}{mRNA})$. $\frac{dY_1}{dt}$ = mean rate of arrival of ribosomes in position 1 - mean rate of arrival of ribosomes in position 2.

We then calculated Y_0 from the equation

$\frac{dY_0}{dt} = mRNA.a_+.G.\left(1-\frac{Y_0}{mRNA}\right) - a_-.Y_0 - b_0.Y_0.(1-\frac{Y_1}{mRNA})$ in steady state and obtained the translation initiation rate:

$$\frac{mRNA.a_+.G.b_0.(1-\frac{Y_1}{mRNA})}{\left(a_+.G + a_- + b_0.(1-\frac{Y_1}{mRNA})\right)}$$

As an example, the maximum translation initiation rate of the capacity monitor when no ribosome already occupied position 1 is

$$\frac{900.0.0001.2500.1.(1-\frac{0}{900})}{(0.0001.2500+200+1.(1-\frac{0}{900}))} = 1.12 \text{ rib.s}^{-1}.$$

Using the model the competition for resources is captured by simulating the production of both the capacity monitor and the synthetic construct proteins within the same model structure, with both specifically parameterised [6].

An important assumption of the original model is that ribosomes move along transcripts one codon at a time and cannot move to the next codon if it is occupied by another ribosome [1]. Thus, ribosome “traffic jams” along the mRNA can be simulated by the model. This helps take into account the impact of changed translation initation rates (different RBS strengths). In our simulations, a high RBS strength with a low γ value will create a traffic jam along the

mRNA, depleting the ribosome pool and so reducing the capacity monitor output (i.e. increasing burden). In this work the validation of the model for accounting for different RBS strengths is tested in Figure 2C where we demonstrate that the burden of constructs with an alternative RBS can be predicted to a relatively good degree ($R^2 = 0.74$) from cell lysate estimations of γ values taken with a different RBS.

Supplementary Note 2: Beta-carotene biosynthesis

In order to quantify the metabolic burden caused by the production of beta-carotene, we assumed that the decrease of capacity was the result of the combined effect of expression burden and metabolic burden. As the predicted burden using cell lysate and our model (Figure 4B) account for expression burden only, we determined the metabolic burden from the difference between the predicted capacity and the measured capacity in *E. coli* strains expressing the original operons (*i.e.* without the inactivating mutation in the *crtE* gene, Figure 4C). This difference was compared to the beta-carotene production in the different strains (Strain OD_{450} – wild type OD_{450} in Supplementary Figure 9B). Most of the strains fit on a linear relationship where more beta-carotene production corresponds to an apparent increase in metabolic burden, demonstrating the relevance of our method (Supplementary Figure 9C). However, as the anticipated metabolic burden could come not only from the production of beta-carotene (the pathway end product) but also from the production of the intermediates geranylgeranyl pyrophosphate, phytoene and lycopene, we expect that using only the beta-carotene production as a proxy underestimates the metabolic burden in most cases. Interestingly, construct B10-2 str (blue dash in Supplementary Figure 9C) stands out as the strain expressing this construct produces more beta-carotene than suggested by the small calculated metabolic burden. B10-2 str is a construct with a predicted strong promoter, weak

RBS1/RBS2/RBS3 and strong RBS4 (Supplementary Figure 9A), which should lead to high expression of CrtY, the last enzyme of the pathway, and low expression of CrtE, CrtB and CrtI (Supplementary Figure 9D). B10-2 str should therefore accumulate a low amount of intermediates and convert metabolites into beta-carotene more efficiently (*i.e.* with less metabolic burden). The appearance of B10-2 str as an outlier in the data reaffirms that the other constructs are likely to be generating unmeasured metabolic burden via intermediate accumulation. Future work could quantify the pathway intermediates in constructs such as these in order to further understand how metabolite conversion and intermediate accumulation leads to *in vivo* metabolic burden, and whether this matches the "predicted – measured normalised capacity" calculation.

1. Algar, R. J. R., Ellis, T. & Stan, G. B. Modelling essential interactions between synthetic genes and their chassis cell. in *Proceedings of the IEEE Conference on Decision and Control* **2015–Feb,** 5437–5444 (2014).
2. Scott, M., Gunderson, C. W., Mateescu, E. M., Zhang, Z. & Hwa, T. Interdependence of Cell Growth and Gene Expression: Origins and Consequences. *Science* **330,** 1099–1102 (2010).
3. Cardinale, S., Joachimiak, M. P. & Arkin, A. P. Effects of genetic variation on the e. coli host-circuit interface. *Cell Reports.* **4,** 231–237 (2013).
4. Marr, A. G. Growth rate of *Escherichia coli*. *Microbiol. Rev.* **55,** 316–333 (1991).
5. Goelzer, A. & Fromion, V. Bacterial growth rate reflects a bottleneck in resource allocation. *Biochim. Biophys. Acta - Gen. Subj.* **1810,** 978–988 (2011).
6. Ceroni, F., Algar, R., Stan, G.-B. & Ellis, T. Quantifying cellular capacity identifies gene expression designs with reduced burden. *Nature Methods* **12,** 415–418 (2015).
