# Supplementary material for: Cell-free prediction of protein expression costs for growing cells
Source: Nat Commun. 2018 Apr 13;9:1457. doi: 10.1038/s41467-018-03970-x (PMC5899134; doi:10.1038/s41467-018-03970-x)
Supplement: Supplementary file 3 — Description of Additional Supplementary Files [file 41467_2018_3970_MOESM3_ESM.pdf]

Description of Additional Supplementary Files

File Name: Supplementary Data 1
Description: Experimental data used to generate the figures in this work
